# The psychosocial and emotional burden of lymphatic filariasis: A systematic review

**DOI:** 10.1371/journal.pntd.0013073

**Published:** 2025-05-08

**Authors:** Jorge Vasconez-Gonzalez, Camila Miño, María de Lourdes Noboa, Andrea Tello-De-la-Torre, Juan S. Izquierdo-Condoy, Esteban Ortiz-Prado

**Affiliations:** 1 One Health Research Group, Faculty of Health Science, Universidad de Las Americas, Quito, Ecuador; 2 Doctoral Program in Occupational Safety and Health, The University of Porto, Porto, Portugal; National Institutes of Health, UNITED STATES OF AMERICA

## Abstract

**Background:**

Lymphatic filariasis (LF) is a neglected tropical disease affecting an estimated 882.5 million people at risk of infection. It is caused by the filarial nematodes *Wuchereria bancrofti*, *Brugia malayi*, and *Brugia timori*, leading to lymphedema, and severe deformation of extremities and resulting in both physical and mental health consequences. Affected individuals often suffer from depression, anxiety, and anger, exacerbated by disability, marginalization, and societal rejection due to their physical disability.

**Methods:**

A systematic review of the literature was conducted following the PRISMA guidelines, using the PubMed, Scopus, and Scielo databases without date restrictions, and including articles in both Spanish and English. The quality of selected articles was evaluated using the Newcastle-Ottawa Quality Assessment Scale and the JBI critical appraisal checklist. Our protocol was registered in PROSPERO under de code: CRD42024537760.

**Results:**

A total of 23 studies were included in this analysis, which identified the emotional impact of LF. Common symptoms include depression, anxiety, frustration, anger, and feelings of inferiority. Physical disability caused by lymphedema led to societal rejection, contributing to the development of these symptoms. We also found that the severity or advancement of the disease correlated with an increased emotional and social impact.

**Conclusions:**

LF significantly impacts the quality of life due to both its physical and emotional consequences. Psychological support for affected individuals is crucial from diagnosis, and the education of transmission and treatment of LF in endemic communities is essential to prevent discrimination and exclusion.

## Author summary

LF is a neglected tropical disease transmitted by vectors among which are *Anopheles, Culex, Aedes*, and *Mansonia* mosquitoes, which is characteristic of becoming chronic and causing deformities, especially in the lower extremities. Beyond physical disabilities, LF imposes significant psychological and social burdens, including stigma, discrimination, anxiety, depression, and even job loss. People affected by this problem experience a great emotional impact due to discrimination, stigmatization, and isolation. With this work, we seek to synthesize information available on the emotional impact and how it affects people infected with LF. The findings from this work will raise awareness about the importance of mental health and encourage research that goes deeper into these aspects, in order to address these patients in a comprehensive way without overlooking their mental health.

## Introduction

Lymphatic filariasis (LF) is a vector-borne neglected tropical disease (NTD) caused by the filarial nematodes *Wuchereria bancrofti, Brugia malayi*, and *Brugia timori* [1]. In 2021, the World Health Organization (WHO) estimated that 882.5 million people in 44 countries across Africa, Southeast Asia, the Pacific, the Caribbean, South America, and the Middle East were at risk of LF [2,3]. The disease burden has a global distribution that primarily corresponds to warm and humid climates where vectors such as *Anopheles*, *Culex*, *Aedes*, and *Mansonia* proliferate and transmit the disease [1]. When these mosquitoes are infected and bite a person, they deposit infective larvae onto the skin that immediately enter the lymphatic system, initiating the parasitic cycle [4].

The disease affects all age groups, and is characterized by asymptomatic damage to the lymphatic system, which then progresses to acute and chronic conditions such as acute adenolymphangitis, sudden fever with lymphadenopathy, or tropical pulmonary eosinophilia [2]. As the disease advances, lymphatic dilation triggers a cascade of pathological events leading to chronic manifestations such as lymphedema and hydrocele. The death of adult worms initiates an acute inflammatory response, resulting in acute lymphadenitis [5].

Patients with acute lymphadenitis may develop lymphedema in the arms, legs, and genitalia, progressing to elephantiasis. Initially, the affected skin is doughy with some indentation, but as inflammation progresses, the tissue becomes firmer, and the indentation disappears. Inflammation extending into the subcutaneous tissue leads to a loss of skin elasticity [5].

Acute filarial lymphadenitis can be exacerbated by secondary bacterial infections, known as acute adenolymphangitis or acute attacks. These episodes are marked by fever, inguinal swelling, pain, tenderness, anorexia, nausea, and vomiting [6]. The long-term deterioration of lymphatic function can display a variety of signs and symptoms depending on the type of filariasis, including lymphedema, hydrocele, elephantiasis, chyluria (milky urine due to the presence of lymph), and chylorrhea (leakage of lymph fluid through the skin). These are among the most severe discomforts reported by patients diagnosed with chronic filariasis [1].

These clinical characteristics of LF have been documented since 38 B.C., when the Romans included them in their encyclopedia “Celsus” [7]. Chronic lymphedema can lead to debilitating complications that, while not fatal, result in long-term disability, social stigma, and significant psychosocial consequences, including anxiety and depression. Additionally, the economic impact is substantial, as many affected individuals are forced to leave their jobs, creating financial strain on their families [8–12]. Despite their severe impact, these aspects have been infrequently studied in medical literature [2,13].

The physical impairment caused by elephantiasis significantly burdens patients’ lives by restricting their mobility. This condition has been extensively documented and reported, including various surgical treatments and therapeutic options aimed at reducing the effects of the disease [4]. Recognizing the intersection between NTDs, stigma, and mental health is essential to ensuring that public health programs adequately address the needs of those affected [14].

In this context, the severe psychological distress related to the social, emotional, and sexual spheres, including couples relationships and sexual life, in the affected population—such as high rates of depression, mental distress, anxiety, lower emotional well-being, poorer social functioning, frequent pain, and suicidal ideation in patients with LF—deserves further attention and exploration [2,15]. Currently, the mental health and socioeconomic burden of LF is often overlooked as a component of morbidity management and disability prevention services, despite the existence of unmet mental health needs [1,4]. While previous reviews have examined the emotional and psychological impact of NTDs, coverage of LF remains limited. In some cases, systematic reviews have included only two to five studies on LF, highlighting a critical research gap [15–17].

To address this issue, we explored the following question: What is the psychosocial impact of LF on affected individuals? This study aims to analyze the primary findings related to the psychosocial and emotional burden of LF, emphasizing its secondary consequences and long-term sequelae. By doing so, we seek to support the advancement of the Global Program to Eliminate LF.

## Methodology

### Study design

We conducted a systematic review, incorporating primary studies such as cross-sectional studies, case-control studies, descriptive observational studies, case reports, and case series. Exclusion criteria included systematic reviews, meta-analyses, narrative reviews, letters to the editor, editorials, and opinion articles. The review adhered to the PRISMA (Preferred Reporting Items for Systematic Reviews and Meta-Analyses) guidelines, which are widely recommended for systematic reviews and meta-analyses. The PRISMA checklist is available in (S1 Table). Our review protocol was registered with PROSPERO under the code CRD42024537760 and is accessible online at: https://www.crd.york.ac.uk/PROSPERO/view/CRD42024537760. All figures accompanying this review were created using the BioRender platform: https://www.biorender.com/

### Search strategies

The literature search was conducted in both Spanish and English to maximize the amount of available information. We reviewed the databases of PubMed, Scopus, and Scielo. To ensure broad coverage, no restrictions were placed on the publication date of included studies. The search was conducted from April 2024 to July 2024.

Additionally, the bibliographies of the articles were reviewed to identify potentially relevant studies. The following search terms were used: ((“lymphatic filariasis” OR “filariasis”) AND (“depression” OR “anxiety” OR “psychological impact” OR “emotional difficulties” OR “social impact”)) in title or abstract. This approach ensured a comprehensive assessment of the psychosocial and emotional burden associated with LF.

### Inclusion criteria

- The inclusion criteria for our systematic review encompassed all manuscripts involving human subjects.
- Studies addressing the emotional impact caused by LF.
- Studies analyzing the social impact of LF.

### Exclusion criterion

The exclusion criteria for our systematic review included studies that compared the impact on mental health of LF with other NTDs. Furthermore, studies evaluating the economic cost or cost-effectiveness of therapies for managing the psychosocial impact of LF were excluded. Additionally, studies that focused on other types of filariasis, such as onchocerciasis, loiasis, mansonellosis, dirofilariasis, or dracunculiasis, were not considered for this review. This ensured that our analysis remained focused on the psychosocial and emotional burden specific to LF.

### Bias assessment

To minimize the risk of bias, the data extraction process was performed independently by JEV, CM, and MLN at different times. In cases where there were discrepancies in collecting information from any primary study, disagreements were resolved through discussion and consensus.

### Data synthesis

We conducted a comprehensive review of all manuscripts meeting our inclusion criteria. Quantitative analysis was performed using the Newcastle-Ottawa Quality Assessment Scale for cohort and case-control studies. For cross-sectional studies, we used the JBI critical appraisal checklist for analytical cross-sectional studies. Case reports and case series were evaluated using the JBI critical appraisal checklists. The studies assessed with these tools were of moderate to high quality. The information from the manuscripts was then organized and synthesized into tables.

## Results

### Literature review

**Fig 1:**
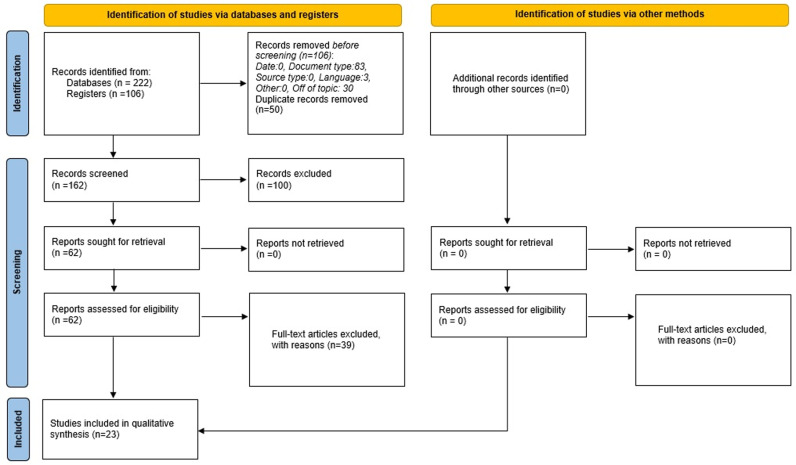
PRISMA flow chart of the studies included in this systematic review.

In adherence to our predefined search strategy and selection criteria, we initially identified a total of 222 studies through our literature search. After removing duplicates and carefully evaluating the abstracts and full manuscripts, we incorporated 23 studies into our analysis (Table 1). Fig 1 provides a detailed visual representation of our selection procedure. Our final sample comprised 1 high-quality cohort study (S2 Table), 3 high-quality case-control studies (S3 Table), and 19 cross-sectional studies, with 10 of high quality and 9 of moderate quality (S4 Table).

**Table 1:** Overview of studies on the association between LF and psychosocial and emotional impact. This table summarizes the authors, year of publication, type of study conducted, the number of participants involved, the primary objective of each study, and the key findings. The studies range from cohort, case-control, and cross-sectional studies analyses, provide information about the emotional impact caused by LF as well as the social impact experienced by affected people.

Author	Year	Type of study	Country	Participants	Objective	Clinical symptom	Result
Abdulmalik and colleagues [8]	2018	Cross-sectional study	Nigeria	69 patients	To evaluate the emotional difficulties and stigma experienced by persons with LF in Plateau State, Nigeria	Lymphoedema	Emotional reactions included feelings of sadness, hopelessness, anger, frustration, worry, and suicidal ideation.
Barrett and colleagues [9]	2023	Cohort study	Malawi	311 adults with filarial lymphoedema	To determine the key mental health indicators affecting people affected by LF lymphoedema by assessing the prevalence of depressive symptoms and QOL.	Lymphoedema	23% (95% CI 18%–29%) reporting mild/moderate depressive symptoms and 31% reporting moderately low/low QOL. Higher depressive symptom scores were associated with high frequency of acute filarial attack episodes.
Eneanya and colleagues [36]	2019	Case and Control	Nigeria	48 patients	To estimate patient numbers and characterize the physical, social and economic impact of LF in in rural Nigeria.	Lymphoedema and hydrocele	40% of all cases reported feeling stigma and were 36 times (95% CI: 5.18–1564.69) more likely to avoid forms of social participation.
Kumari and colleagues [24]	2005	Cross-sectional study	India	174 patients	To measure the severity levels of seven health states of LF in the physical and psychosocial domains of health from the perspective of patients and medical experts, using a 7-domain 5-level.	Lymphoedema and hydrocele	People with higher grades of lymphoedema and hydrocele had more severe psychosocial problems than physical ones
Kwarteng and colleagues [37]	2023	Cross-sectional study	Ghana	72 participants	To assist in refining a large qualitative study (currently underway) that seeks to integrate culturally appropriate LF interventions into current LF control programs in Ghana.	NA	79.2% of the affected individuals responded that they felt ashamed or stigmatized, as a result of the disease.
Mangeard-Lourme and colleagues [26]	2020	Cross-sectional study	India	2559 patients	To assess the scale for depression and anxiety among people affected by leprosy and LF seeking medical care in India, and the main risks associated with poor mental health	Lymphoedema and hydrocele	50% of people affected LF reported experiences of depression and anxiety.
Martindale and colleagues [27]	2014	Cross-sectional study	Malawi	69 patients	To assess the severity of lymphoedema, the physical restrictions, and socio-economic impact on affected individuals living in an endemic community in Malawi	Lymphoedema	Lymphoedema cases were most affected by pain/discomfort and anxiety/depression
Obindo and colleagues [18]	2017	Cross-sectional study	Nigeria	98 participants	To determine the prevalence and severity of depression among individuals with physical disfigurement from LF in Plateau State, Nigeria.	Lymphoedema	20% of the respondents met criteria for depression, with the following proportions based on severity: Mild (42.1%), Moderate (31.6%), and Severe (26.3%).
Paramanik and colleagues [33]	2020	Cross-sectional study	India	1193 school children	To assess the LF epidemiology as well as awareness about the disease among school children in the rural areas of Bankura district.	Lymphoedema and hydrocele	66.21% were found to be anxious about the fate of their LF problems.
Perera and colleagues [38]	2007	Cross-sectional study	Sri Lanka	60 participants	To increase understanding of how this vulnerable, neglected group can be helped.	Lymphoedema and hydrocele	The stigma attached to the condition caused social isolation and emotional distress, and delayed diagnosis and treatment, resulting in undue advancement of the disease.
Person and colleagues [29]	2007	Cross-sectional study	Dominican Republic	56 women	To obtain an in-depth perspective and understanding of the psychosocial and health consequences associated with lymphedema of the leg among women in the Dominican Republic.	Lymphoedema	Women in our study described a spectrum of consequences associated with their lymphedema but physical, functional, and psychological limitations.
Person and colleagues [30]	2008	Cross-sectional study	Dominican Republic	56 women	To understand better the psychological states that accompany the physical and functional limitations of disease, as well as the coping strategies that women use to ameliorate negative psychological states.	Lymphoedema	Women across all stages of lymphedema described not only the detrimental psychological effect of lymphedema but also positive adaptive coping strategies to help them with the psychological sequelae.
Person and colleagues [31]	2007	Cross-sectional study	Dominican Republic	28 women	To identify specific factors associated with intact or disrupted social connectedness among Dominican women with chronic filarial lymphedema.	Lymphoedema	Women described complications of aging, disability, reduced social networks, and inability to adhere to cultural scripts as contributing to disrupted social connectedness.
Person and colleagues [32]	2009	Cross-sectional study	Dominican Republic and Ghana.	104 women	To investigate how women with lymphedema from two different cultures experience stigma and its consequences	Lymphoedema	Women described a spectrum of enacted, perceived, and internalized stigma experiences, such as being criticized and isolated by the community, health providers, and even by friends and relatives.
Richard and colleagues [11]	2007	Cross-sectional study	Togo	188 participants	To describe the results of a baseline survey of a lymphoedema morbidity management program in Togo.	Lymphoedema	Patients felt avoided by their family (17%) and their community (36%).
Seekles and colleagues [19]	2023	Cross-sectional study	Democratic Republic of the Congo	118 participants	To develop and evaluate a community-based mental health intervention for people affected by skin NTDs.	Lymphoedema	In total, 58.3% of men and 80.0% of women screened positive for major depressive disorder
Suma and colleagues [25]	2013	Cross-sectional study	India	127 participants	To assess the perceptions, practices, and socio-psychological problems of 127 patients with brugian LF.	Lymphoedema	Depression and loss of job opportunities were common in the study population. Patients also complained that the disease eroded their standing in the community and diminished their prospects of marriage.
Thapa and colleagues [20]	2023	Cross-sectional study	Nepal	102 NTD-affected persons (70 leprosy and 32 LF)	To examine the relationship between stigma, depression, and QOL among people affected by leprosy and LF in Nepal.	N/A	The mean scores were 21.8 ± 4.4 for QOL, 6.6 ± 5.6 for depression, and 3.0 ± 2.8 for stigma. Almost 17% reported the prevalence of depression symptoms.
Tyrell [28]	2012	Cross-sectional study	Guyana	50 participants	To assess the socioeconomic impact of LF in Guyana.	Lymphoedema and hydrocele	40% indicated that some of the changes included anxiety, cost of living, embarrassment, and separation from spouses.
Udo and colleagues [21]	2024	Cross-sectional study	Nigeria	people affected by NTDs (leprosy, 32; LF, 16)	To determine the rate of depression and anxiety, respectively, among people affected by skin NTDs.	Lymphoedema	100% of those affected with LF have anxiety and 75% have depression.
Wijesinghe and Wickremasinghe [23]	2010	Case and control study	Sri Lanka	141 filarial lymphoedema patients and 128 healthy people	To describe and quantify the QOL of patients with chronic filarial lymphoedema.	Lymphoedema	Chronic lymphedema has a significant negative impact on the QOL as perceived by affected patients.
Wijesinghe and colleagues [12]	2007	Cross-sectional study	Sri Lanka	413 participants	To describe the physical disability and psychosocial impact associated with chronic lymphoedema in patients attending filariasis clinics.	Lymphoedema	25% and 6% reported having problems interacting with the community and family, respectively and 8.7% felt that they were rejected by society.
Wijesinghe and Wickremasinghe [22]	2012	Case and control study	Sri Lanka	141 filarial lymphedema patients and 128 healthy people	To describe and quantify the physical, psychological, and social aspects of the QOL in patients with chronic filarial lymphedema.	Lymphoedema	F-36 revealed that patients experienced poorer physical functioning, more role limitations because of physical health problems, less emotional well-being, poorer social functioning.

LF, lymphatic filariasis; NTDs, neglected tropical diseases; QOL, quality of life.

### Tools used for the analysis of emotional and psychosocial impact

Various tools have been employed to evaluate the mental and psychosocial distress caused by LF. Depression was frequently assessed using the Patient Health Questionnaire (PHQ-9), a nine-item Likert-scale tool aligned with the DSM criteria for major depressive disorder [9,18–21]. Patients scoring 5 or higher on the PHQ-9 were further interviewed using the depression module of the Composite International Diagnostic Interview (CIDI) [18]. Anxiety was evaluated using the 7-item Generalized Anxiety Disorder Assessment [19,21], and the Duke Anxiety-Depression (DUKE-AD) scale, a seven-question standardized tool, was also applied [11].

Quality of life was assessed with several instruments, including the WHOQOL-BREF, a 26-item tool measuring physical health, psychological health, social relationships, and environment [19]. Some studies used the WHOQOL-100, which provides scores for 24 facets, six domains, and overall quality of life and health perception [22,23]. Thapa and colleagues utilized the eight-item EUROHIS-QOL scale, a shorter version of WHOQOL-BREF, to measure global quality of life, physical health, personal psychosocial health, and environmental health [20]. Barrett et al. employed the Lymphatic Filariasis-Specific Quality of Life Questionnaire (LFSQQ) [9].

Stigma was analyzed using the five-question Stigma Indicator-Affected People (5-QSI-AP) scale, which evaluates areas such as concealment, avoidance, pity, and shame [20]. Other tools included the GHQ-30 questionnaire, the SF-36 (36 items), and the extended EuroQoL (7D5L), which assesses physical, mental, and social health impacts [22,24].

### Emotional impact of LF

**Fig 2:**
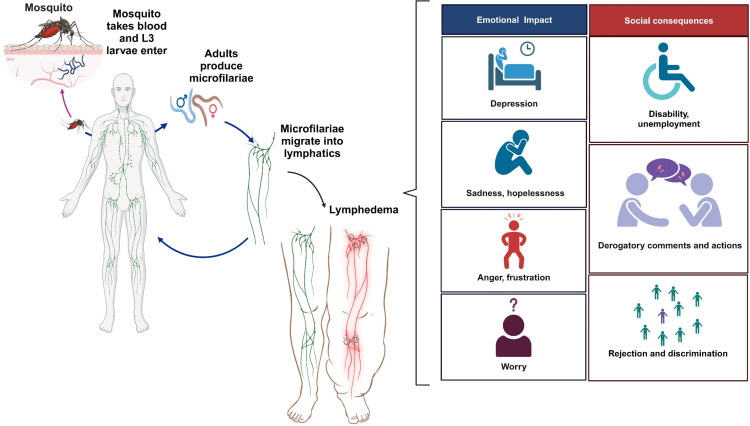
Emotional impact of LF (created with BioRender).

Several studies have analyzed the emotional impact of LF (Fig 2), with depression and anxiety being the most common outcomes, most of the studies come from Nigeria (*n* = 4), India (*n* = 4), Sri Lanka (*n* = 4), and the Dominican Republic (*n* = 4). Qualitative analyses involving 69 and 127 patients revealed emotional problems such as sadness, hopelessness, anger, frustration, worry, depression, feelings of inferiority, and suicidal ideation [8,25]. A cohort study involving 311 adults found that 23% (95% CI: 18%–29%) reported mild to moderate depressive symptoms, with higher scores associated with frequent acute filarial attacks [9].

In India, a cross-sectional study involving 2,559 LF cases reported that 50% experienced depression and anxiety. Obindo and colleagues found that among LF patients, 42.1% had mild depression, 31.6% moderate, and 26.3% severe [18]. Similarly, Udo and colleagues found that 56% of LF patients suffered from depression, with varying severities [21,26].

Factors such as the inability to heal, work, support one’s family, carry out usual activities, wear clothes and shoes, and participate in social activities contribute to the development of depression and anxiety in people with LF [27]. Notably, 58.3% of men and 80.0% of women tested positive for major depressive disorder using the Patient Health Questionnaire (PHQ-9) [19]. Thapa and colleagues conducted a cross-sectional study and found a mean PHQ-9 score of 5.9 ± 5.7, with 46.9% of participants classified as normal, 37.5% as mild, 3.1% as moderate, and 12.5% as severe [20]. Wijesinghe and colleagues, in their case-control study, used the Short Form 36 (SF-36) tool, which revealed that cases experienced lower emotional well-being [22]. The 30-item General Health Questionnaire (GHQ-30) showed that the mental well-being of controls (better than usual; 67.2%) was significantly better than that of cases (36.2%, *P* < .001).

In terms of anxiety, research in Nigeria found that 100% of LF patients experienced anxiety. Tyrell’s study found that 40% of LF patients had anxiety accompanied by shame. Generalized anxiety disorder symptoms were observed in 54.8% of men and 62.2% of women [19,28].

Four studies focused exclusively on women [29–32], revealing that psychological consequences can outweigh physical ones, with disfigurement leading to feelings of shame, sadness, depression, loneliness, and suicidal ideation [29,31]. Person and colleagues found that in women affected with LF, shame, and guilt were the most frequent feelings caused by lymphedema, in addition to the fact that at some point they begin to experience depression; another study carried out in the Dominican Republic and Ghana revealed in both locations that women commonly experience feelings of fear, guilt, shame, sadness, depression, and diminished self-worth as a result of lymphedema caused by filariasis [30,32].

A study in India involving 1,193 school children found that 66.21% were anxious about their filarial problems. Anxiety was higher among girls (84%) compared to boys (62.50%) due to societal and medical ignorance. Additionally, 55.86% of children felt ashamed of revealing the disease, with girls (88%) being more affected than boys (49.17%) [33].

### Impact of the severity of lymphedema and subsequent hydrocele

**Fig 3:**
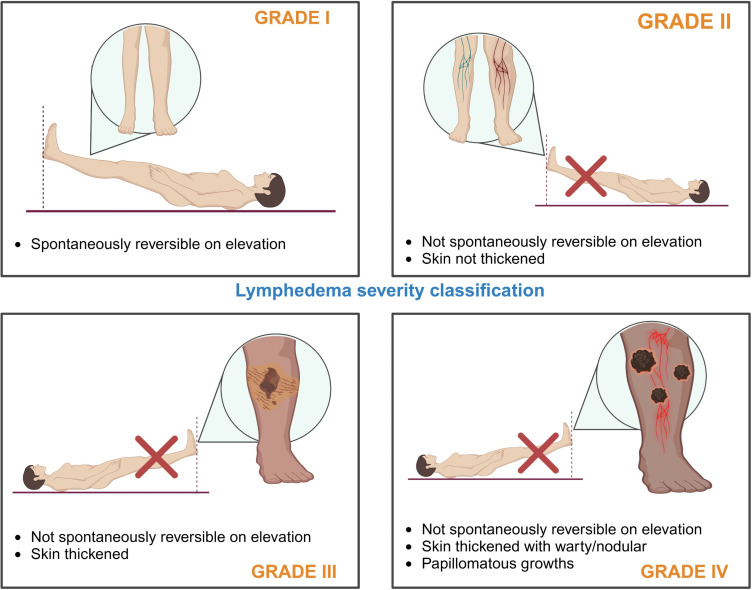
Classification of Lymphedema Severity (created with BioRender).

Several studies have analyzed the emotional impact of the severity of lymphedema caused by LF [12,22–24]. All of these studies classified the level of lymphedema according to standard criteria [34] (Fig 3). It has been found that even in low grades of lymphedema, such as grade II, there are already physical difficulties, which worsen as the grade of lymphedema increases. Wijesinghe and colleagues observed that more than half of the patients in their study lost or left their jobs due to lymphedema, indicating a significant social impact [12]. The greater the degree of lymphedema, the more problems patients experienced attending social gatherings and leisure activities. A quarter of the participants had issues interacting with the community, leading them to isolate themselves and withdraw from society, generating feelings of anger, bitterness, and depression. Additionally, 37% of the participants reported that edema made them shy, depressed, and intensely worried about the progression of the disease to elephantiasis [12].

The severity of the condition increases as the disease progresses, being greater in grade IV patients. Factors such as physical disability, loss of income, and dependence on others generate feelings of shame, anxiety, and depression. As a result of the physical disability, disturbing thoughts lead patients to lose interest in their activities, causing distraction and an inability to concentrate [24]. Furthermore, due to the stigma and shame associated with edema, affected individuals tend to avoid social activities. From grade II onward, patients experience mild anxiety and depression accompanied by feelings of anger. In grade III, moderate pain and physical disabilities cause moderate anxiety and cognitive problems. Unlike grade I, mental and social health areas are significantly affected [24].

Chronic lymphedema has a significant negative impact on the quality of life as perceived by affected patients. The duration of lymphedema also plays an important role. It was observed that patients who had suffered from lymphedema for more than 31 years had a significantly lower quality of life around physical security compared to patients who had it for a shorter time. The spiritual sphere—encompassing how individuals seek meaning, purpose, transcendence, and their connection to the sacred [35]—is profoundly affected in the most severe stages of lymphedema. Many affected individuals perceive their condition as a divine curse, further exacerbating their emotional distress and sense of hopelessness [23].

Kumari and colleagues analyzed the impact of hydrocele resulting from LF and observed that people with grade II hydrocele experienced mild problems in the mental and social spheres, including embarrassment associated with the physical disability, which also caused anxiety and moderate depression [24]. In contrast, those affected with grade I hydrocele did not experience any impact on the mental and social areas.

### Stigma, discrimination, and social rejection

Stigma, discrimination, and social rejection are frequent problems suffered by people affected by LF, as analyzed in various studies. Research conducted on women affected by LF in the Dominican Republic revealed that perceived stigma included discrimination, fear, and uncertainty of being marginalized, ashamed, or rejected. These women believed that due to the physical disability of their legs, they would be judged and belittled. Patients reported being victims of derogatory comments and actions from community members, family members, and even health providers, which caused them shame [29,30,32]. A study conducted in Nigeria revealed that 40% of cases reported feeling some type of stigma from people inside and outside their community. Similarly, a study in Ghana found that 79.2% of affected individuals felt ashamed or stigmatized [36,37]. In a case-control study, 70% of the controls admitted to stigmatizing people with lymphedema [36].

Regarding social rejection, patients reported having problems with their community and even with family members. Wijesinghe et al. found that 25% of patients had issues getting along with the community, and 8.7% felt completely rejected. Additionally, 5.8% revealed having problems interacting with their own families [12]. Other studies have shown that 36% of people with LF are avoided by community members and 17% by their own families [11]. However, a study conducted in Malawi with 69 participants observed that 93% reported no problems with social participation [27].

Women affected by this disease reported decreased social interactions and exhaustion of existing relationships. Loss of functionality, shame due to disfigurement, and lack of appropriate footwear contributed to social isolation [30,32]. The patients are not the only ones affected; families with a member suffering from LF tend to be marginalized by their neighbors. Children were often prohibited from interacting with such families and from accepting water or food from them. If they did, they were punished or even beaten [25].

## Discussion

To the best of our knowledge, this research represents the first systematic review focused exclusively on the emotional and psychosocial impact caused by LF. While other reviews have addressed the emotional impact and mental health in NTDs, the focus on LF has been very limited. For example, a review published in 2021 included only 4 studies on LF, and another in 2022 included 6 studies on stigma in LF, while for leprosy, they included 45 studies [14,15].

Comorbidity associated with mental illness caused by NTDs is a seriously underestimated dimension of the global burden of many of these diseases [39]. GBD studies on mental disorders do not specifically mention NTDs, even though WHO data indicate that 1 billion people suffer from some type of NTD, many of which leave long-term sequelae associated with anxiety, depression, and stigma [40,41]. NTDs produce distress and mental illness through mechanisms such as pain, disability, disease stage and duration, stigma, discrimination, and restriction or exclusion from social activities [14]. The most reported mental disorders in NTDs are depression and anxiety [37–41], which can lead to more serious mental, neurological, and social problems, including substance use as a coping mechanism or thoughts of self-harm or suicide [41].

LF, like many other NTDs, is considered a disease of poverty associated with high levels of disability due to changes in the lower and upper extremities, breast, or scrotum, resulting in lymphedema or hydrocele. Studies in Nigeria revealed that 20% of people with LF have depression, compared to 3.1%–5.2% in unaffected individuals. According to the WHO, depression nearly doubles the total burden of disease in people with LF, from 2.78 to 5.09 million disability-adjusted life years (DALYs) [15,18,41]. The burden of depressive illness in patients with filariasis is 5.09 million DALYs, with an additional 229,537 DALYs attributable to their caregivers [39]. Obindo et al. noted that a history of mental illness is a risk factor for recurrence or the development of new illness; their study found that people with LF and a history of mental illness are almost 41 times more likely to be depressed than those without such a history [18]. Self-esteem also plays an important role, with high self-esteem protecting against the development of depression in this group [18,42].

**Fig 4:**
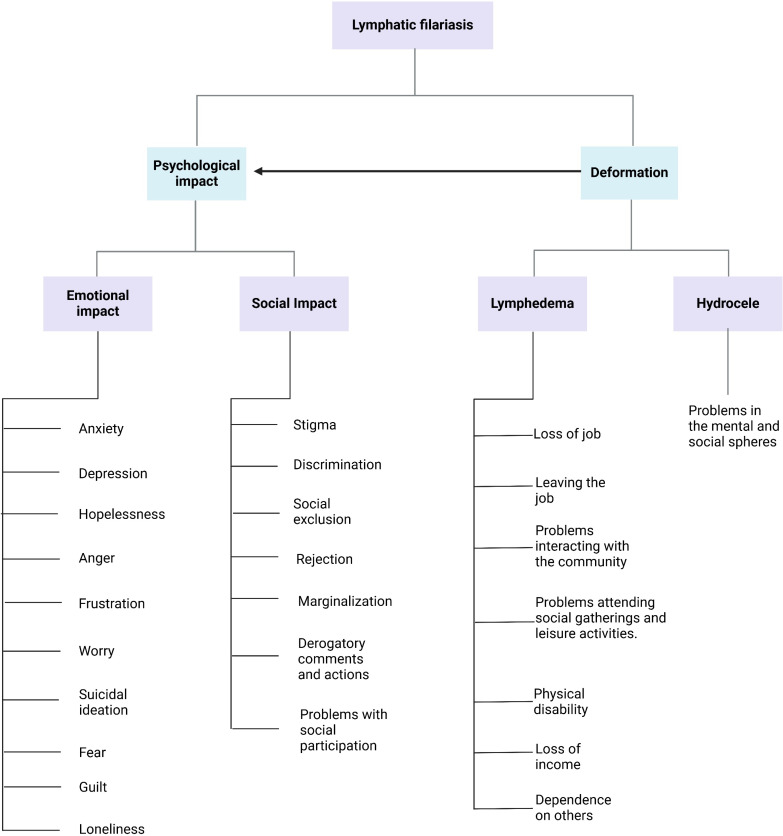
Emotional impact and social consequences of LF (created with BioRender).

This review identifies that LF produces feelings of inferiority, anger, frustration, sadness, hopelessness, depression, anxiety, and suicidal ideation as a result of lymphedema-induced physical disability (Fig 4). The resulting disability often leads to unemployment, and affected individuals suffer rejection and discrimination from society and sometimes from their families, exacerbating these feelings. The social rejection experienced by these individuals has been described as causing high levels of disability, preventing them from completing their education, obtaining employment, or maintaining jobs [8].

The duration of the disease is associated with a greater prevalence of depression, and stigma and anxiety are more severe in the advanced stages of the disease [18,36]. In this review, we found that depression is present from grade II lymphedema onwards, with mild anxiety evolving to moderate in grade III; in grade II hydrocele, mild mental health problems begin to appear; this is likely because they cannot hide their condition [8]. Abdulmalik and colleagues observed that individuals with minimal inflammation managed to lead as normal a life as possible [8]. However, Berret and colleagues highlighted that while stigma was reported less frequently among individuals with mild lymphedema—particularly those with less visible disabilities and older participants—it remained a persistent issue [10].

Due to the growing concern about mental health in patients with LF and other NTDs, there is a need to create a global normative guide to incorporate mental health support for affected individuals, such as the WHO guide on mental health and NTDs or the WHO NTD Roadmap 2021–2030 [43]. Different studies have demonstrated the benefits of psychological support. Agarwal and colleagues showed that after 3 months of psychological support, the stigma level decreased from 30.3 to 24 (*p* < 0.001); high mental well-being increased from 0% to 13.3% (*p* < 0.001); and moderate to severe depression decreased from 88% to 47% (*p* < 0.001) [44]. Mol and colleagues, in their study, observed that after 2 months of psychological support, stigma was significantly reduced [45]. A study evaluating a holistic care package observed significant improvements in disability scores, quality of life, depression, stigma, discrimination, and social support after 12 months [46]. Aggithaya et al. demonstrated that a community-based integrative self-care approach led to marked improvements in mobility, self-care, daily activities, pain and discomfort, and social relationships (*p* < 0.01) [47]. Additionally, community-based lymphedema treatment programs have been shown to significantly reduce disability scores (*p* < 0.0001) [48].

### Limitations

This review has several limitations, including the heterogeneity of the studies used. Not all studies employed tools to evaluate depressive symptoms, such as the PHQ-9, or emotional health, such as the GHQ-30 or the SF-36. Many studies used techniques such as focus groups, making it difficult to accurately measure and evaluate the presence of emotional problems. Additionally, several studies were limited to qualitative analyses and did not provide data on the proportion of people with LF who suffer from emotional problems or data to classify the most frequent issues. Few studies have evaluated how different degrees of edema and hydrocele influence emotional health.

Language restrictions might have excluded pertinent studies, limiting the evidence base. The exclusion of gray literature and theses, whether undergraduate or doctoral, may have contributed to the loss of important information. Another significant limitation is the limited information on how the level of education can contribute to the development of emotional problems or to the stigmatization of people affected by LF.

## Conclusion

LF significantly impacts those who suffer from it, not only due to the physical disability caused by lymphedema and hydrocele but also because of the various emotional and mental health problems it can cause. These range from feelings of anger, sadness, and frustration to issues like depression, anxiety, and even suicidal ideation. Additionally, individuals with LF face discrimination, marginalization, and social rejection from their communities and even their own families. This social discrimination means they often cannot obtain or retain employment and are unable to participate in social events, further contributing to the development of depression, anxiety, and low self-esteem.

Given these challenges, psychological counseling is vital from the moment the diagnosis is made. It is also important to educate families and communities about LF to prevent discrimination and marginalization. Misconceptions, such as the belief that LF is a contagious disease or a curse, often drive this discrimination. Governments and relevant authorities must take measures to address these issues by promoting projects that provide medical and psychological care for these individuals, especially in areas with limited access to health services.

## Supporting information

S1 Table PRISMA Checklist. (DOCX)

S2 Table NEWCASTLE—OTTAWA QUALITY ASSESSMENT SCALE for cohort studies. (DOCX)

S3 Table NEWCASTLE—OTTAWA QUALITY ASSESSMENT SCALE for case-control studies. (DOCX)

S4 Table JBI critical appraisal checklist for analytical cross-sectional studies. (DOCX)
