# Supplementary material for: The psychosocial and emotional burden of lymphatic filariasis: A systematic review
Source: PLoS Negl Trop Dis. 2025 May 8;19(5):e0013073. doi: 10.1371/journal.pntd.0013073 (PMC12084059; doi:10.1371/journal.pntd.0013073)
Supplement: S2 Table — (DOCX) [file pntd.0013073.s002.docx]

**S2 Table :** NEWCASTLE - OTTAWA QUALITY ASSESSMENT SCALE

| **Study** | **1** | **2** | **3** | **4** | **5** | **6** | **7** | **8** | **Score** |
| --- | --- | --- | --- | --- | --- | --- | --- | --- | --- |
| Barrett et al. [9] | * |  | * | * | ** | * | * | * | 8 |
|  |  |  |  |  |  |  |  |  |  |

**Note:** A study can receive a maximum of one star for each item numbered within the Selection and Result categories. A maximum of two stars can be awarded for comparability.

**Selection**

1. Representativeness of the exposed court.

2. Selection of the unexposed court.

3. Exposure determination.

4. Demonstration that the current outcome of interest was not present at baseline.

**Comparability**

5. Cohort comparability based on design or analysis.

**Results**

6. Evaluation of the result.

7. Was the follow-up long enough for the results to occur?

8. Adequacy of cohort follow-up.

**Interpretation**

Good quality: 3 or 4 stars in the selection domain and 1 or 2 stars in the comparability domain and 2 or 3 stars in the outcome/exposure domain.

Acceptable quality: 2 stars in the selection domain and 1 or 2 stars in the comparability domain and 2 or 3 stars in the outcome/exposure domain.

Poor quality: 0 or 1 star in the selection domain or 0 stars in the

comparability domain or 0 or 1 stars in the outcome/ exposure domain
