# Supplementary material for: The psychosocial and emotional burden of lymphatic filariasis: A systematic review
Source: PLoS Negl Trop Dis. 2025 May 8;19(5):e0013073. doi: 10.1371/journal.pntd.0013073 (PMC12084059; doi:10.1371/journal.pntd.0013073)
Supplement: S3 Table — (DOCX) [file pntd.0013073.s003.docx]

**S3 Table:** NEWCASTLE - OTTAWA QUALITY ASSESSMENT SCALE

| **Study** | **1** | **2** | **3** | **4** | **5** | **6** | **7** | **8** | **9** | **10** | **Score** |
| --- | --- | --- | --- | --- | --- | --- | --- | --- | --- | --- | --- |
| Eneanya et al. [37] | * |  | * | * | * | * | * | * |  | * | 8 |
| Wijesinghe & Wickremasinghe [24] | * |  | * | * | * | * | * | * |  | * | 8 |
| Wijesinghe & Wickremasinghe [23] | * |  | * | * | * | * | * | * |  | * | 8 |

**Is the Case Definition Adequate?**

1. Requires some independent validation (e.g. >1 person/record/time/process to

extract information, or reference to primary record source such as x-rays or

medical/hospital records)

2. Record linkage (e.g. ICD codes in database) or self-report with no reference to

primary record

3. No description

**Representativeness of the Cases**

4. All eligible cases with outcome of interest over a defined period of time, all cases

in a defined catchment area, all cases in a defined hospital or clinic, group of

hospitals, health maintenance organization, or an appropriate sample of those

cases (e.g. random sample)

5. Not satisfying requirements in part (a), or not stated.

**Selection of Controls**

6. Community controls (i.e. same community as cases and would be cases if had

outcome)

7. Hospital controls, within same community as cases (i.e. not another city) but

derived from a hospitalized population

8. No description

**4 Definition of Controls**

9. If cases are first occurrence of outcome, then it must explicitly state that controls

have no history of this outcome. If cases have new (not necessarily first)

occurrence of outcome, then controls with previous occurrences of outcome of

interest should not be excluded.

10. No mention of history of outcome
