# Supplementary material for: The psychosocial and emotional burden of lymphatic filariasis: A systematic review
Source: PLoS Negl Trop Dis. 2025 May 8;19(5):e0013073. doi: 10.1371/journal.pntd.0013073 (PMC12084059; doi:10.1371/journal.pntd.0013073)
Supplement: S4 Table — (DOCX) [file pntd.0013073.s004.docx]

**S4 Table:** JBI critical appraisal checklist for analytical cross-sectional studies

| **Study** | **1** | **2** | **3** | **4** | **5** | **6** | **7** | **8** | **Score** |
| --- | --- | --- | --- | --- | --- | --- | --- | --- | --- |
| Abdulmalik et al. [8] | YES | YES | YES | YES | YES | NO | YES | YES | 7 |
| Kumari et al. [25] | YES | YES | YES | YES | YES | NO | YES | YES | 7 |
| Kwarteng et al. [38] | YES | YES | YES | YES | YES | YES | YES | YES | 8 |
| Mangeard-Lourme et al. [27] | YES | YES | YES | YES | YES | YES | YES | YES | 8 |
| Martindale et al. [28] | YES | YES | YES | YES | YES | YES | YES | YES | 8 |
| Obindo et al. [19] | YES | YES | YES | YES | YES | NO | YES | YES | 7 |
| Paramanik et al. [34] | YES | YES | YES | YES | YES | NO | YES | YES | 7 |
| Perera et al. [39] | YES | YES | YES | YES | YES | YES | YES | YES | 8 |
| Person et al. [30] | YES | YES | YES | YES | YES | NO | YES | YES | 7 |
| Person et al. [31] | YES | YES | YES | YES | YES | NO | YES | YES | 7 |
| Person et al. [32] | YES | YES | YES | YES | NO | NO | YES | YES | 6 |
| Person et al. [33] | YES | YES | YES | YES | YES | YES | YES | YES | 8 |
| Richard et al. [11] | YES | YES | YES | YES | YES | YES | YES | YES | 8 |
| Seekles et al [20] | YES | YES | YES | YES | YES | YES | YES | YES | 8 |
| Suma et al. [26] | YES | YES | YES | YES | YES | YES | YES | YES | 8 |
| Thapa et al. [21] | YES | YES | YES | YES | YES | YES | YES | YES | 8 |
| Tyrell [29] | YES | YES | YES | YES | NO | NO | YES | YES | 6 |
| Udo et al. [22] | YES | YES | YES | YES | YES | YES | YES | YES | 8 |
| Wijesinghe et al. [12] |  |  |  |  |  |  |  |  |  |

1. Were the criteria for inclusion in the sample clearly defined?

2. Were the study subjects and setting described in detail?

3. Was the exposure measured in a valid and reliable way?

4. Were standard and objective criteria used to measure the condition?

5.Were confounding factors identified?

6. Were strategies established to deal with confounding factors?

7. Were the outcomes measured in a valid and reliable way?

8. Was an appropriate statistical analysis used?

Quality score were categorized into three groups: Low: 1-4, Moderate: 5-7, and High:8
